# Survey of the Knowledge, Attitudes and Practice towards Antibiotic Use among Prospective Antibiotic Prescribers in Serbia

**DOI:** 10.3390/antibiotics11081084

**Published:** 2022-08-10

**Authors:** Olga Horvat, Ana Tomas Petrović, Milica Paut Kusturica, Dragica Bukumirić, Bojana Jovančević, Zorana Kovačević

**Affiliations:** 1 Faculty of Medicine, Department of Pharmacology Toxicology and Clinical Pharmacology, University of Novi Sad, 21000 Novi Sad, Serbia; 2 Institute of Public Health “Dr Milan Jovanovic Batut”, 11000 Belgrade, Serbia; 3 Faculty of Agriculture, Department of Veterinary Medicine, University of Novi Sad, 21000 Novi Sad, Serbia

**Keywords:** antibiotics, self-medication, students, habits

## Abstract

The complex issue of antibacterial resistance (ABR) requires actions taken with the One Health approach, involving both human and veterinarian medicine. It can spread from animals to humans through the food chain or through direct contact. Health profession students, as the future antibiotic providers, can greatly impact antibiotic-related issues in the future. The study was conducted to evaluate knowledge, attitudes and practice of future antibiotic prescribers in relation to judicious use of antibiotics. This cross-sectional, questionnaire-based study was performed on 400 students of health professions who were allowed to prescribe antibiotics of the University of Novi Sad, Serbia. Students of medicine and students of dentistry showed a significantly higher knowledge score compared to students of veterinary medicine (*p* = 0.001). Multivariate regression identified predictors of adequate antibiotic knowledge: being a female student (B = 0.571; *p* = 0.020), higher grade average (B = 1.204; *p* = 0.001), students of medicine (B = 0.802; *p* = 0.006) and dentistry (B = 0.769; *p* = 0.026), and students who used a complete package of antibiotics during the last infection (B = 0.974; *p* = 0.001) or for the period recommended by the doctor (B = 1.964; *p* = 0.001). Out of the total sample, self-medication was reported among 42.8% of students. The identified predictors of self-medication were: more frequent (B = 0.587; *p* = 0.001) and irregular (B = 0.719; *p* = 0.007) antibiotic use, taking antibiotics until symptoms disappeared (B = 2.142; *p* = 0.001) or until the bottle was finished (B = 1.010; *p* = 0.001) during the last infection. It seems prudent to reevaluate the educational curricula regarding antibiotic use and ABR of prospective prescribers in Serbia.

## 1. Introduction

Although antibacterial resistance is one of the most serious global public health threats in this century, antibacterial resistance (ABR) represents at the moment the major problem, both for the high rates of resistance observed in bacteria that cause common infections and for the complexity of the consequences of ABR [1].

The One Health concept has appeared to go hand in hand with the issue of antibiotic resistance as the most comprehensive and global solution, since there are many ways in which ABR can be transferred between humans and animals, via close contact, through the food chain or indirectly via the environment [2,3]

Better managing the problem of ABR includes taking steps to preserve the continued effectiveness of existing antibacterials such as trying to eliminate their inappropriate use, especially to reduce the number of unnecessary prescriptions, since new antibiotics coming to the market have not kept pace with the increasing need for improvements in antibiotic treatment [4]. What is particularly important is that antibiotics used in human medicine are to some extent the same as those used in veterinary medicine, and evidence is emerging that antibiotics critical for human health are being used among veterinary professionals [5,6]. Besides the widely implemented antimicrobial stewardship programs for improving the antimicrobial prescription in human and veterinary practice, the World Health Organization advocates to implement strategies that allow the next generation of doctors to be better prepared to appropriately use antibiotics and combat bacterial resistance [4,7,8].

Whereas several studies have tried to measure the knowledge, attitude and practice (KAP) of prospective doctors towards antibiotics, the existing literature is mainly focused on antibiotic prescribing practices rather than on KAP about antibiotic consumption. In addition, many of them show a lack of KAP on the importance of judicious use of antibiotics and optimal antibiotic prescription practices [9,10,11,12,13].

Serbia belongs to a group of European countries with the highest rates of resistance, as well as a high antibiotic consumption rate [14,15]. Based on a 2020 report by the Central Asian and European Surveillance of Antimicrobial Resistance (CAESAR) network, which includes 18 non-European Union countries and areas, high percentages of resistance in *P. aeruginosa*, *Acinetobacter* spp. and *E. faeciumi* in Serbia are concerning, in addition to the moderately high resistance for third-generation cephalosporins, aminoglycosides and fluoroquinolones in *E. coli* and for penicillin and macrolides in *S. pneumoniae* [14]. Furthermore, Serbia with the consumption of 31.57 DDD per 1000 inhabitants per day is together with Greece (33.85) and Turkey (38.18) among the three countries with the highest overall antibiotic consumption in Europe according to the WHO Europe Antimicrobial Medicines Consumption Network report for the period 2016–2018 [15]. In 2019, following the resolution of the United Nations by all countries, the Republic of Serbia formulated its National Antimicrobial Resistance Control Program for 2019–2023, where among other strategies, special attention is given on increasing awareness among those who prescribe antimicrobials [16]. In Serbia, the medical education of prospective medical doctors, dentists and veterinarians offers specialized curricula including pharmacology and microbiology, but not courses of antimicrobial use and resistance. Therefore, the aim of this study was to evaluate the knowledge, attitude and practice of students of medicine, dentistry and veterinary medicine who will be the only prospective prescribers of antibiotics in Serbia.

## 2. Results

### 2.1. Sociodemographic and Academic Characteristics

Almost two-thirds (62.5%) of the total surveyed students were female (Table 1). The mean age of the subjects was 23.80 ± 1.53 years. The observed students of the fifth and sixth year of medicine were on average older compared to the students of the fourth and fifth years of dentistry and veterinary medicine. Students of medicine (89.5%) and students of dentistry (92%) had a higher average grade (8.00–10.00) compared to students of veterinary medicine (61%) (*p* <0.005). There was no statistically significant difference between these groups of students in relation to place of residence (*p* = 0.007), number of visits to general practitioners in the past 12 months (*p* = 0.107) and in relation to whether they have a family member who is a healthcare worker (*p* = 0.162).

**Figure 1:**
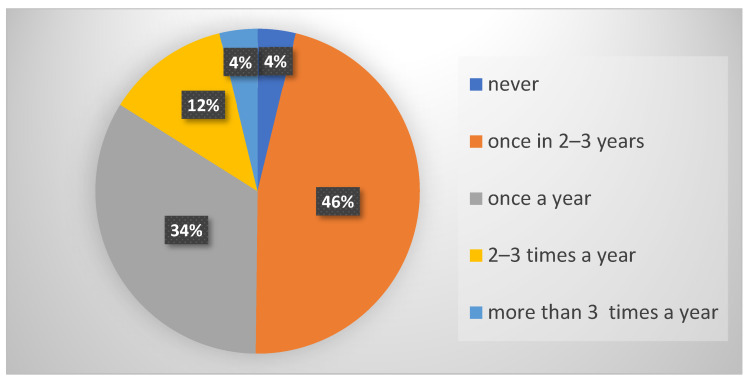
Frequency of taking antibiotics in all surveyed students.

There were no statistically significant differences among the examined groups of students in relation to the frequency of taking antibiotics (*p* = 0.318). The majority of surveyed students (46%) claimed to take antibiotics once every 2–3 years (Figure 1).

### 2.2. Students’ Knowledge Regarding the Antibiotics Use

Respondents showed good knowledge regarding the use of antibiotics (Table 2). The percentage of correct answers was higher than 80% in 9 of the 12 proposed claims. The lowest percentage of correct answers was recorded for the statement “Antibiotics are taken until the whole package is consumed”. Of the 400 students surveyed, only 64% gave the correct answer. Of the 400 respondents, 250 (62.5%) showed adequate knowledge while 150 (37.5%) had inadequate knowledge. The median knowledge of all surveyed students was 11 (interquartile range, 5–11). Students of medicine and students of dentistry had a significantly higher knowledge score compared to students of veterinary medicine (*p* < 0.001). The median knowledge of students of medicine was 11 (interquarter range, 10–12), the median knowledge of students of dentistry was 11 (interquarter range, 10–12), while the median knowledge of student students of veterinary medicine was 10 (interquarter range, 9–11).

### 2.3. Practice and Attitudes of Respondents toward Antibiotics Use

A lower share of students of medicine and students of dentistry compared to students of veterinary medicine irregularly used antibiotics prescribed by doctors (*p* < 0.032) (Table 3). To the question “What do you do when you think the antibiotics you are taking are ineffective?”, 72% of students of medicine and 65% of students of dentistry stated that they continue to take antibiotics recommended by a doctor. The share of students of veterinary medicine who stated the same, as well as those who reported that they stop taking antibiotics and go to the doctor, was the same (43%) (*p* < 0.001). The share of students of medicine (74.5%) who used antibiotics as recommended by their doctors during their last infection was higher compared to students of dentistry (61%) and students of veterinary medicine (63%) (*p* < 0.047).

Among sociodemographic and academic characteristics, and responses related to student practice and attitudes, six predictors of adequate knowledge were statistically significant in univariate models (*p* < 0.05). The multivariate logistic regression model that predicts adequate knowledge showed that female students have 1.8 times higher chance of adequate knowledge (OR = 1.8; B = 0.571; *p* = 0.020) than male students (Table 4). Students of medicine (OR = 2.2; B = 0.802; *p* = 0.006) and students of dentistry (OR = 2.2; B = 0.769; *p* = 0.026) have a 2.2 times higher chance of adequate knowledge compared to students V as a reference category. Students with an average grade of 8 and higher have an over 3 times higher chance of adequate knowledge (OR = 3.3; B = 1.204; *p* < 0.001) compared to students with a lower average grade. Students who took antibiotics during the last infection for a period the doctor recommended (OR = 7.1; B = 1.964; *p* < 0.001) and those who took antibiotics until the whole package was finished have a better chance of adequate knowledge (OR = 2.6; B = 0.974; *p* < 0.001) compared to those students who used antibiotics until their symptoms relieved as a reference category (7.1 times and 2.6 times the chance, respectively).

M—students of medicine; D—students of dentistry; V—students of veterinary medicine. The multivariate logistic regression model included 3 predictors of self-medication, which were analyzed on 400 subjects (of whom 171 reported self-medication). The tendency for practicing self-medication significantly increased with more frequent use of antibiotics (*p* < 0.001) as shown in Table 5. Students who irregularly took antibiotics prescribed by their doctor had twice the chance of self-medication (OR = 2.1; B = 0.719; *p* = 0.007). Students who took antibiotics in the last infection until they finished the whole package (OR = 2.7; B = 1.010; *p* < 0.001) or until symptoms disappeared (OR = 8.5; B = 2.142; *p* < 0.001) were more likely prone to self-medication in relation to those students who used antibiotics for the period the doctor recommended as a reference category (2.1 and 8.5 times higher chance, respectively).

#### Reasons for Self-Medication with Antibiotics

**Figure 2:**
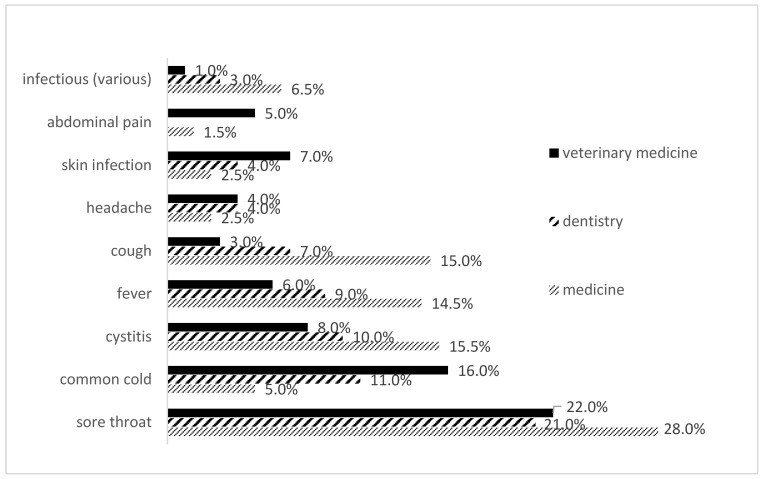
Indications listed as reasons for self-medication with antibiotics among medical, dental and veterinary medicine students in 2019 in Serbia.

The most common reason for self-medication was sore throat (28% for students of medicine, 22% for students of veterinary medicine and 21% for students of dentistry). In addition to this cause for practicing self-medication, students of veterinary medicine and students of dentistry were most likely to list common cold as a reason for self-medication, whereas students of medicine were most prone to self-medication in case of cystitis and cough (15.5% and 15.0%, respectively) (Figure 2).

## 3. Discussion

No detailed study assessing KAP of Serbian students of medicine, dentistry and veterinary medicine about antibiotics has to our knowledge been published so far. This is also a unique study that simultaneously surveys students on three different courses, gaining valuable insight into this topic among prospective prescribers.

Our findings showed how the healthcare profession students had a fairly good knowledge about antibiotics, although the percentage of students with adequate knowledge was only slightly higher than among the general population in Serbia (62.5% vs. 61.6%, respectively) [17]. More than 80% of the sample answered correctly to all the knowledge-related items administered, with two important exceptions. Indeed, almost 40% of the sample stated that antibiotics should be taken until the whole package is consumed and around 25% of students reported that taking antibacterial drugs twice a day means after waking up and before going to bed. Previous studies have found similar misconceptions regarding adherence to antibiotic regimens among the general population in Serbia, but we found a small reduction in these beliefs among students who were at the final stages of their course [17]. Given the crucial role that healthcare professionals play as daily communicators with the general public, we think it is important that students completing their healthcare course are aware of and able to communicate certain basic concepts, for example, that inappropriate use of antibiotics could harm both humans and animals and increase the prevalence of resistant strains. In addition, our results agree with other findings about knowledge of antibiotic use reported among students in China Ethiopia, Poland and Italy, as well as among adults in Jordan [18,19,20,21,22]. Regarding the Balkan region, single-center studies have found gaps in knowledge on antibiotic use among students of medicine and pharmacy in Croatia and among adults in Albania, Kosovo and the Republic of Macedonia [23,24,25,26]. However, some studies showed that knowledge of antibiotic use was moderate to poor among medical and veterinary students [13,27,28].

Although 81.3% of the total sample stated antibiotics cannot be used for the treatment of common cold, it is surprising that this percentage was significantly lower among students of veterinary medicine (63%) compared to students of medicine (85.1%) and dentistry (91%) (*p* < 0.001). Similar results have been observed among students of veterinary medicine in Bangladesh, where about 30% did not know that antibiotics were ineffective against viruses and only 56.79% believed antibiotics could not speed up the recovery of common cold. On the contrary, most students (92%) on selected healthcare courses in the United Kingdom agreed that most coughs, colds and sore throats get better on their own without the need for antibiotics, but 25% of students D still thought that antibiotics were effective against colds [29]. Similar findings have been reported among students of medicine by many studies, which could indicate their greater familiarity with the indications [21,30,31]. Considering the impact of veterinary students’ future professional practices on ABR, it would be relevant to conduct similar surveys in this group, because a large proportion of these students were unaware that antibiotics do not cure viral infections such as the common cold. Evidence of improper antibiotic behavior patterns were also found among future prescribers of antibiotics in our survey. Whereas only 21% of students of medicine and students of dentistry reported irregular antibiotic use, among students of veterinary medicine 34% stated this practice, which is near to the percentage recorded among the general population in Serbia (40%) [17]. There are many conditions in which stopping antibiotics upon resolution of symptoms increases the risk of the patient experiencing a relapse with resistant pathogenic bacteria [32]. Interestingly, in our study, students of dentistry and veterinary medicine more frequently used antibiotics only until their symptoms resolved compared to students of medicine (*p* = 0.047). This practice, besides leading to relapse of infection and lack of treatment efficacy, also contributes to the emergence and spread of antibiotics resistance, a very current problem in Serbia [14,33].

It is additionally worth noting that students of dentistry (61%) and students of veterinary medicine (63%) showed decreased willingness to use antibiotics during their last infection as advised by the doctor compared to students of medicine (74.5%) (*p* = 0.047). It may be a symptom of lack of trust in colleagues and lack of humility, even though it is increasingly apparent that One Health approaches are essential to tackling ABR [17,34]. As with prescribing doctors and veterinarians, who often lay the blame for ABR and poor behaviors on other groups, students of veterinary medicine in the study of Golding et al. (2022) also consider their own (future) prescribing to be less of a contributor to ABR than that of other prescribers [35]. Furthermore, the results of the present study are encouraging if compared to the Serbian general population. As much as 7.5% of the prospective healthcare professionals in our study used leftover antibiotics during their last infections, which is considerably lower than among the general population (17%) [17]. This practice was higher among medical students in Italy and Turkey and veterinary students in Portugal and Bangladesh [12,21,36,37]. An effective strategy to combat incorrect antibiotics use consists of continuing education to improve knowledge and awareness of both professionals and the general population [21,38,39].

Some of the most interesting contributions of the present study are the conclusions derived from the logistic regression analysis conducted to determine predictors of adequate knowledge among prospective prescribers of antibiotics.

Firstly, this study revealed a sex difference in KAP toward knowledge on antibiotic use. Female students showed almost a two times higher chance of adequate knowledge than male students. This is similar to a study performed in Italy among medical, dental and nursing students as well as among university students in the United Arab Emirates, whereas this difference was not recorded among students in Mumbai [21,40,41] The fact that gender plays a vital role in antibiotics is well-documented [42]. In the survey among the outpatients in England and Wales, females were prescribed higher antibiotics than males, and they suffered the most from antibiotic misuse as they showed lack of knowledge regarding the proper use of antibiotics in comparison to their male counterparts [43]. Secondly, students of medicine and dentistry have a more than two times higher chance of adequate knowledge compared to students of veterinary medicine.

This agrees with other findings about adequate knowledge on antibiotic use among prospective healthcare professionals reported in the literature [9,18,19,21]. On the other hand, studies among final-year veterinary students reported knowledge gaps on antibiotic use [12,31]. There is some evidence that, as would be expected, medical and veterinary curricula do have the desired effect of improving knowledge about responsible use of antimicrobials [44,45]. Although general practitioners and dentists are responsible for nearly 80% of all antibiotic prescriptions written, efforts should also be intensified for greater exposure to the prudent use of antibiotics during clinical teaching in the final year of veterinary school, which may lead to greater awareness and perceptions of ABR [46].

Our results showed that the most common reason for self-medication with antibiotics reported by all students‘ groups was sore throat. However, the most reported condition for students of veterinary medicine and students of dentistry was common cold, whereas students of medicine were most likely to practice self-medication with antibiotics in case of cystitis and cough. This is comparable with the results of some previous studies. For example, a recent study performed in Saudi Arabia among future prescribers showed that sore throat was the most common reported symptom, followed by fever and cough [47]. In addition, studies conducted in Nepal, India, Sri Lanka and China all showed that the common cold was the most common medical condition for which medical students self-medicated with antibiotics [18,48,49]. Slightly different results were observed in the Sudanese study, where the most common symptoms treated by medical students with non-prescription antibiotics were acute respiratory tract symptoms, cough and common cold [50]. Furthermore, the findings from the UK study showed that 25% of dentistry students were of the opinion that antibiotics were effective against colds [51].

Logistic regression models also showed interesting results in terms of identifying the most relevant factors influencing self-medication with antibiotics of interviewed students. Students who more frequently used antibiotics and who irregularly took them showed a higher probability for practicing self-medication. Similarly, among 1042 medical students from two universities in Saudi Arabia, 97.2% of them had used antibiotics in the previous year, whereas half of them reported self-medication [52]. Several scientists agree that it is essential to provide more information about antibiotics and the possible adverse effects of their improper use because such information helps decrease the frequency of use and encourages the correct use of these drugs. However, it has also been documented that knowledge does not always correlate with practice [53].

Finally, the present study also highlighted that students who used antibiotics during the last infection until the bottle was finished or those who reported cessation of antibiotic use after their symptoms resolved were less likely to have adequate knowledge and were more likely to self-medicate than those who reported antibiotic usage for the period advised by the doctor. These findings are in line with those obtained in other studies, which points to the need for more education on antibiotics use in degree courses, especially as students are future clinicians, teachers and researchers who will be at the forefront in educating the general public [9,35,37].

The strength of the present study is that this is the first survey exploring the knowledge, attitude and practice toward antibiotics among prospective prescribers in Serbia, and is thus a very valuable contribution to the current literature. Another strength of the present study that should be highlighted is a large sample size. However, despite the large sample size, interpretation of the findings should be done with caution due to the self-administered questionnaire and the possibility of the participants to over- or under-report socially desirable behaviors. The second limitation that should be mentioned is that the study was conducted at only one university in Serbia, and thus the findings may not be with certainty extrapolated to the national level. Notwithstanding these limitations, we have succeeded in identifying some key knowledge gaps among the medical students who participated in our study and provided useful findings for public health policymakers in Serbia.

## 4. Materials and Methods

### 4.1. Study Design

The study was conducted in January and February 2019 at the University of Novi Sad, one of the largest Serbian universities. The study population comprised a total of 400 final-year students in the 2018/2019 academic year of the integrated academic study programs of medicine (200), dentistry (100) and veterinary medicine (100). The selected groups of students are prospective healthcare professionals who will be permitted to prescribe antibiotics in the Republic of Serbia. The sample size, calculated based on the estimate that 81.3% of medical science students practice self-medication, was 234, with a relative accuracy of 10% and a confidence interval of 95% [16,17]. The sample size was intentionally exceeded, taking into account the possibility of excluding respondents from the study, withdrawal and the need for subgroup analysis. After signing the informed consent, the respondents filled out the questionnaire. The research was approved by the Ethical Committee of the Faculty of Medicine in Novi Sad (approval number 01-39/290/1).

### 4.2. Questionnaire Design

The questionnaire used in this study was based on the questionnaire of Buke et al. [38] (Annex 1). Necessary modifications have been made to provide correct answers to questions and claims. The content, comprehension, readability and appearance of the questionnaire were previously tested on 30 students at the University of Novi Sad who were not included in the final analyses.

The questionnaire was divided into three parts. The first part, which contained 8 questions, included demographic characteristics of respondents and some general questions (age, gender, year of study, field of study, average grade, place of residence, number of visits to a general practitioner in the past 12 months, is anyone in the family a healthcare worker). The second part referred to the respondents’ knowledge regarding the use of antibiotics. This part contained 12 statements with the possibility of answering with true or false. The questions were about the conditions that require the use of antibiotics, how to start antibiotic treatment, the duration of antibiotic therapy, the meaning of the claim “take twice a day”, and whether frequent and inadequate use of antibiotics can be harmful. The knowledge score was determined by giving one point for each correct answer and the maximum score was 12.

The third part, which consisted of 15 questions with the possibility of answering yes or no, as well as multiple-choice answers, referred to the attitudes and practice of students toward antibiotics. This part was focused on the prophylactic use of antibiotics, self-medication with antibiotics, adherence to the antibiotic therapy, on whose recommendation the students take antibiotics, and how they used them during the previous infection.

### 4.3. Data Analysis

Descriptive and comparative statistical analysis of the results was performed with IBMSPSS Statistics 22 (IBM Corporation, Armonk, NY, USA) software. In addition to descriptive statistical methods, measurements of central tendency (arithmetic mean, median), measures of variability (standard deviation) and frequency were also used. Two variables were created, antibiotic knowledge status (adequate–inadequate) and self-medication (yes–no). The categories of knowledge were determined according to the median recent knowledge of the total sample (11, interquartile range 5–12). Respondents were grouped into those with adequate (knowledge score ≥11) and those with inadequate knowledge score (knowledge score ≤11). Self-medication was analyzed based on questions from the third part of the questionnaire. The variable self-medication was formed by combining the answers to the questions “Have you ever used antibiotics to avoid getting sick” (those who answered “yes”), “Have you ever started taking antibiotics on your own” (those who answered “yes”) and “The last time you used antibiotic, who recommended it to you?”(those who answered any of the following—“I used previously used antibiotic or advised by a friend; I used antibiotics previously prescribed by my doctor; I asked a pharmacist and used the antibiotics recommended”).

For testing statistical hypotheses, the χ^2^ test, Mann-Whitney U test, analysis of variance with post hoc test, the Kruskal-Wallis test and Fisher test of exact probability were used. All *p* values less than 0.05 were considered significant.

The association of the students’ characteristics with adequate knowledge about antibiotics and self-medication was primarily evaluated using univariate logistic regression. Multivariate logistic regression also included predictors of all variables with *p* < 0.05 in univariate analysis. The results are presented as odds ratio (OR) with 95% confidence interval (CI). All *p* values less than 0.05 were considered significant.

Although students of medicine and students of dentistry demonstrated a higher level of knowledge about antibiotic use than students of veterinary medicine, as far as attitude and practices regarding ABR and usage are concerned, there is a significant need for improvement among all three groups of future prescribers. It is a reasonable starting point as a way to tackle the problem from both human and animal welfare standpoints. Therefore, our study provides an important insight regarding their knowledge, attitude and practice, which can be considered, in order to plan for an effective undergraduate curriculum regarding antibiotic resistance and usage. The solution to this problem requires a concerted One Health approach to mitigate future risks to humans, animals and the environment.

## Figures and Tables

**Table 1:** Socio-demographic and academic characteristics of students of medicine (M), dentistry (D) and veterinary medicine (V) of the University of Novi Sad.

		M		D		V		*χ* ^2^	*p*	Total	
		*n*	%	*n*	%	*n*	%			*n*	%
Gender	Male	75	37.5	26	26	49	49	11.285	0.004	150	37.5
	Female	125	62.5	74	74	51	51			250	62.5
Average grade	6.00–7.99	21	10.5	8	8	39	39	46.031	0.001	68	17
	8.00–10.00	179	89.5	92	92	61	61			332	83
Place of living	with parents	64	32	43	43	38	38	17.568	0.007	145	36.3
	in university dormitories	33	16.5	7	7	14	14			54	13.5
	in rented apartments	67	33.5	32	32	43	43			142	35.5
	in own apartment	36	18	18	18	5	5			59	14.8
Number of visits to GP in the last 12 months	None	109	54.5	46	46	39	39.8	4.467	0.107	194	48.7
	1–4	82	41	52	52	57	58.2			191	48
	5–10	7	3.5	2	2	2	2			11	2.8
	>10	2	1	0	0	0	0			2	0.5
Having a family member who is healthcare worker	Yes	70	35	38	38	26	26	3.636	0.162	134	33.5
	No	130	65	62	62	74	74			266	66.5

**Table 2:** Knowledge of students of medicine (M), dentistry (D) and veterinary medicine (V) use antibiotic use.

	M		D		V		*χ* ^2^	*p*
	*n*	%	*n*	%	*n*	%		
Antibiotics are used to decrease fever	174	87	91	91	75	75	11.234	0.004
Antibiotics are used to decrease pain	190	95	95	95	88	88	5.839	0.054
Antibiotics are used to overcome malaise and fatigue	197	98.5	97	97	97	97	1.023	0.501
Antibiotics are used for common cold	171	85.5	91	91	63	63	30.474	<0.001
Antibiotic treatment begins with an antibiotic found at home in order not to waste time	180	90	95	95	94	94.9	3.551	0.169
Antibiotic treatment is started after a visit to doctor and with a doctor’s prescription	194	97	97	97	97	97	0.125	1
Antibiotic treatment is started when it is advised by a pharmacist	171	85.5	79	79	67	67	13.88	<0.001
Antibiotic is used until the symptoms disappear	167	83.5	88	88	75	75	6.13	0.047
Antibiotic is used until the bottle finishes	136	68	65	65	55	55	4.98	0.084
Antibiotic is used as long as the doctor prescribes	194	97	97	97	96	96	0.239	0.933
Taking the medicine twice a day means after waking up and before going to bed	136	81.5	80	80	64	64	12.231	0.002
Frequent and improper use of antibiotics is harmful and dangerous	194	97	98	98	95	95	1.511	0.501

**Table 3:** Attitudes and practice of students of medicine (M), dentistry (D) and veterinary medicine (V) about antibiotic use.

	M		D		V		*χ* ^2^	*p*
	*n*	%	*n*	%	*n*	%		
Have you ever used antibiotics in order not to get ill?								
No	200	100	100	100	100	100		1
Have you ever started antibiotics on your own when you got ill?								
Yes	75	37.5	37	37	32	32	0.933	0.627
Have you ever used antibiotics prescribed by the doctor irregularly?								
Yes	42	21	21	21	34	34	6.9	0.032
What do you do when you think that antibiotic you are taking is not effective?								
I stop taking it and go to the doctor	36	18	22	22	43	43	32.444	<0.001
I stop taking it and go to another doctor	8	4	1	1	6	6		
I use it for the recommended period	144	72	65	65	43	43		
Other	12	6	12	12	8	8		
How did you use antibiotics during your last infection?								
Until the bottle is finished	38	19	27	27	22	22	9.634	0.047
Until the symptoms disappeared	13	6.5	12	12	15	15		
As advised by the doctor	149	74.5	61	61	63	63		
How did you get antibiotics during your last infection?								
I used the antibiotic previously used or as advised by my friends or relatives	5	2.5	8	8	5	5	8.354	0.392
I used the antibiotic previously prescribed by my doctor	17	8.5	4	4	9	9		
I visited my doctor and used the prescribed antibiotic	144	72	73	73	69	69		
I asked the pharmacist and used the antibiotic recommended	8	4	4	4	2	2		
I do not remember	26	13	11	11	15	15		

**Table 4:** Multivariate logistic regression with adequate knowledge of antibiotics as a dependent variable.

Independent Variables	B	*p*	OR	95% Confidence Intervals	
				Lower	Upper
Male gender	Reference category				
Female gender	0.571	0.02	1.8	1.095	0.571
V	Reference category				
M	0.082	0.006	2.23	1.25	3.96
D	0.769	0.026	2.16	1.09	4.24
How often do you take antibiotics?	−0.214	0.132	0.087	0.611	1.07
Average grade	1.204	<0.001	3.332	1.77	6.28
Have you ever used antibiotics prescribed by the doctor irregularly?	−0.376	0.174	0.687	0.39	1.18
How did you use antibiotics during your last infection?					
Until the symptoms disappeared	Reference category				
As advised by the doctor	1.964	<0.001	7.13	3.01	16.83
Until the bottle is finished	0.974	0.036	2.649	1.06	6.58

**Table 5:** Multivariate logistic regression model with self-medication as a dependent variable.

Independent Variables	B	*p*	OR	95% Confidence Intervals	
				Lower	Upper
How often do you take antibiotics?	0.587	<0.001	1.798	1.35	2.38
Have you ever used antibiotics prescribed by the doctor irregularly?	0.719	0.007	2.052	1.21	3.47
As advised by the doctor	Reference category				
Until the bottle is finished	1.01	<0.001	2.746	1.62	4.65
Until the symptoms disappeared	2.142	<0.001	8.514	3.33	21.76

## Data Availability

All data are available upon a reasonable request from the authors.
